# Beyond the Concept of Winter-Summer Leaves of Mediterranean Seasonal Dimorphic Species

**DOI:** 10.3389/fpls.2019.00696

**Published:** 2019-05-31

**Authors:** Giacomo Puglielli

**Affiliations:** Chair of Biodiversity and Nature Tourism, Estonian University of Life Sciences, Tartu, Estonia

**Keywords:** leaf cohorts, intraindividual variability, trait-environment relationships, climate change, seasonality

## Introduction

One of the dominant growth forms in Mediterranean ecosystems is represented by seasonally dimorphic (semi-deciduous) species. Seasonal dimorphism has been long considered as a key adaptation to the seasonality of the Mediterranean type of climate for shrub species inhabiting the Mediterranean Basin (batha, phrygana and maquis shrublands, Orshan, 1964; Margaris, 1975, 1977; Margaris et al., 1984; Christodoulakis et al., 1990; Gratani and Crescente, 1997; Kyparissis et al., 1997; Díaz Barradas et al., 1999), Californian chaparral (Westman, 1981; Mooney and Miller, 1985), Chilean matorral (Montenegro et al., 1979) and South African Cape karroid shrublands (Westman, 1981). The main feature of seasonal dimorphic species is the development of two well-defined leaf cohorts during a growing season, the so-called “winter” and “summer” leaves (Westman, 1981, Nilsen et al., 1986; Aronne and De Micco, 2001), hereafter referred as WL and SL, respectively.

Nevertheless, there is evidence that seasonal dimorphism can be flexible (Orshan and Zand, 1962; Orshan, 1964; Westman, 1981; Kyparissis et al., 1997; Palacio et al., 2006, 2008; Puglielli et al., 2017, in press; Puglielli and Varone, 2018), so that the extent of differences between WL and SL, as well as the number of cohorts during a growing season, depend on species and environmental conditions at a given site. For instance, the existence of an intermediate step between WL and SL (generally called spring leaves) has been suggested in previous studies on seasonal dimorphic species (e.g., Palacio et al., 2006; de Dato et al., 2013; Puglielli et al., in press). At any rate, the flexibility of seasonal dimorphism can result in leaves to be formed under different prevailing environmental conditions due to the seasonality of the Mediterranean climate and significant changes in leaf traits are expected between leaves formed in different periods during a given growing season (Morales et al., 2014; Niinemets, 2014). The presence of multiple leaf cohorts during a growing season should be reflected by a great degree of intraindividual variability in leaf traits, but this has never been analyzed in an explicit way.

## Intraindividual Variability in Seasonal Dimorphic Species: Differences Between and Within Leaf Cohorts

Seasonal dimorphic species indeed display a high variability of several functional traits during one growing season. By calculating coefficient of variations (CV) for leaf length, width, area (LA), leaf mass area (LMA), thickness and nitrogen content on area basis for four *Cistus* spp. from Correia and Ascensão (2017), average trait variability spans from 25 to 51% during an entire growing season depending on the species. Similarly, Palacio et al. (2008) by analyzing seasonal changes in leaf dry matter content (LDMC) in four Mediterranean seasonal dimorphic species, found a 2- to 3-fold variation at the intraspecific level and such seasonal changes were higher than interspecific differences. They also found that different cohorts were simultaneously present in different types of shoots occupying different positions along the heteroblastic series, stressing the relationship between leaf trait variability and the presence of multiple cohorts.

Comparable trends over a season can be also observed for the Mediterranean seasonal dimorphic *Halimium halimifolium* growing in four sites differing in water availability (Díaz Barradas et al., 1999). Calculating CVs for LA, leaf dry mass (DM) and LMA, CVs range between 103–143% for LA, 25–30% for DM, and 6–28% for LMA, in line with the previous data on *Cistus* spp. However, it is interesting to point out that LMA variability in Díaz Barradas et al. (1999) depends on site. If we consider LMA as an indicator of shifts in leaf level strategies between leaf cohorts, such dependence of leaf traits variability on the site reflects the possible convergence/divergence between leaf cohorts as affected by environmental conditions (see previous section and Palacio et al., 2006).

**Figure 1:**
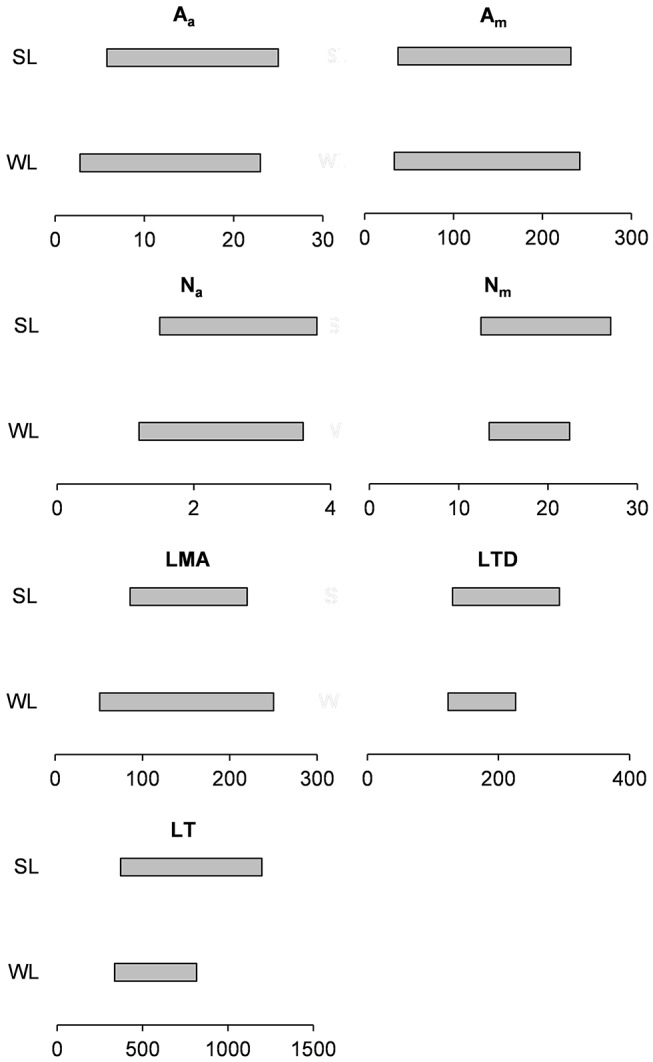
Range of variability in some leaf functional traits of winter (WL) summer leaves (SL) of 9 *Cistus* spp. Modified from Puglielli Varone 2018. A_a_ (μmol CO_2_ m^−2^ s^−1^), photosynthesis per unit of leaf area; A_m_ (nmol CO_2_ g^−1^ s^−1^), photosynthesis per unit of leaf mass; N_a_ (g m^−2^), leaf nitrogen content per unit of leaf area; N_m_ (g g^−1^), leaf nitrogen content per unit of leaf mass; LMA (g m^−2^), leaf mass area; LTD (mg g^−1^), leaf tissue density; LT (μm), leaf thickness.

Recently, it has been also demonstrated (Puglielli and Varone, 2018) that there is a great inherent variability of leaf functional traits in WL and SL of 9 *Cistus* spp. (43% of the species belonging to the genus) across the Mediterranean Basin. Also, the authors found that the considered traits had overlapped ranges of trait variation between leaf cohorts (Figure 1). This confirms that leaves belonging to different leaf cohorts can converge in some circumstances in terms of mean values of leaf traits as a result of the high degree of variability of climatic factors within the Mediterranean Basin.

Surprisingly, a similar magnitude of traits variation can be found even in the medium-term (i.e., few months) within WL and SL in *Cistus* spp. (Correia and Ascensão, 2017) for key leaf traits, such as leaf area (LA) and leaf mass area (LMA). For two *Cistus* spp. for example, CV for LA is, on average, 69 and 63% for WL and SL, while those for LMA are 30 and 5% compared to those calculated over a growing season of 71% for LA and 33% for LMA. Similarly, a degree of variability for LA up to 40 and 60% can be found within WL and SL belonging to the same plant in five seasonal dimorphic species of Californian coastal sage in Westman (1981), with average coefficient of variations across species of 20 and 42% for WL and SL, respectively. Such great variability results in a 3- to 21-fold difference in LA between WL and SL, underscoring that intraindividual differences between these leaf cohorts can be more or less pronounced even at the level of a single plant.

At the within leaf cohort level, Puglielli et al. (2017) demonstrated that WL (sampled between November and April) of three *Cistus* spp. have different slopes for the relationship LMA-photosynthesis per unit of leaf mass (A_m_) during the transition from winter to spring. This result is particularly relevant since A_m_ characterizes the biochemical capacity of single cells and is the key player in the worldwide trade-off between the physiological and structural characteristics of leaves (Westoby et al., 2013; Niinemets et al., 2015; Tosens et al., 2016). So, changes of bivariate relationships LMA- A_m_ indeed highlight leaf specific adaptations to particular environmental conditions (Wright et al., 2004). Significant differences in slopes of bivariate relationships between functional traits with varying environmental conditions have been further confirmed during an entire growing season (from November to July, Puglielli et al., in press).

## Functional Role of Intraindividual Variability in Seasonal Dimorphic Species: Insights from *Cistus* spp.

Overall, the previous analysis supports the existence of a seasonal spectrum of leaf traits variation which can be unlikely represented by a simple “winter-summer” classification of seasonal dimorphic species leaf cohorts. In other words, the degree of intraindividual variability in leaf traits of seasonal dimorphic species can be underestimated by analyzing only differences between leaf cohorts that mostly picture broad differences between leaves formed at the extremes of a given growing season. Accordingly, more detailed leaf flushing analyses on seasonal dimorphic species have already revealed contrasting patterns across species in different climatic conditions which were not always consistent with seasonal dimorphism itself (Palacio et al., 2006).

At any rate, the functional role of intrandividual variability in seasonal dimorphic species along a growing season has not yet been clarified. Analysis of the changes in leaf traits covariation patterns between leaf cohorts of *Cistus* spp. (see Puglielli and Varone, 2018; Puglielli et al., in press) can provide some insights on the functional relevance of such variability. A different acclimation of leaves to different environmental conditions, as expected for different leaf cohorts, should be highlighted by consistent differences in slopes and/or intercepts of bivariate relationships between leaf functional traits (i.e., “Shift 2 scenario” *sensu* Wright et al., 2001). Nevertheless, this was not always the case for a broad comparison of WL vs. SL (Puglielli and Varone, 2018), nor for leaves diachronically formed along a given growing season (Puglielli et al., in press). Interestingly, seasonal changes in slopes of the bivariate relationships can parallel changes in resource-acquisition and use strategies *sensu* (Pierce et al., 2013; Puglielli et al., in press), partially providing a mechanistic explanation of a seasonal spectrum of traits variation. So, *Cistus* spp., and likely other seasonal dimorphic species, can shift from a relatively lower (more stress tolerant) to a relatively higher (more competitive) return strategy in the medium-term by adjusting trade-off rates between functional traits to the prevailing environmental conditions in which the leaves are formed. Such behavior also stresses a previous hypothesis from Niinemets (2014, but see also Puglielli and Varone, 2018) that leaf flushing strategy (i.e., the number of leaf cohorts) may be an important driver altering the leaf economic spectrum (Wright et al., 2004).

Herrera et al. (2015), but see also Herrera (2009), argued that interpreting the adaptive role of within-plant variation in functional traits requires an independence between intraindividual variability in a given trait and trait means. In a similar fashion, this is what Puglielli et al. (in press) somewhat found: the above mentioned slopes of bivariate relationships followed seasonal changes of the climatic factors according to temperature response curves while mean values did not.

Thus, the degree of intraindividual variability in leaf traits along a growing season is linked to seasonal dimorphic species ability to adjust in the medium-term leaf physiology and morphology (and their relationships) to changing environmental conditions. This is particularly important in view of the unpredictability of the Mediterranean climate, since such fine adjustments can enable seasonal dimorphic species to efficiently track favorable (and mostly variable) climatic conditions in order to maximize whole-plant performance. In line with this statement, intraindividual variation in functional leaf traits has been proposed to be a means to optimize exploitation of environmental variation both in time and space and to enhance whole-plant photosynthetic performance (Givnish, 1988; Mulkey et al., 1992; Hollinger, 1996; Winn, 1996, 1999; Osada et al., 2014; Herrera et al., 2015).

Nevertheless, one can argue that intraindividual modifications in both leaf structure and function as well as their relationships along a growing season can be a costly strategy in species characterized by a relatively short leaf life span, such as seasonal dimorphic species. However, leaves formed under different environmental conditions can mostly coexist in the canopy until the onset of summer drought. So, I argue that changes in leaf traits trade-off rates over an entire growing season can balance the rate of return of the investment made in leaf stiffness and/or carbon gain in the long term. This would make species able to maintain the costs of leaf economics' even if the single leaves are characterized by a relatively short leaf life span.

## Concluding Remarks

A great degree of intraindividual variability in seasonal dimorphic species and its link with environmental changes undermine the efficacy of a simple “winter-summer” leaf classification. In fact, when accounting for this source of variability, which magnitude can be comparable to interspecific one (Iannetta et al., 2007; Boucher et al., 2013; Laforest-Lapointe et al., 2014; Medrano et al., 2014; Mitchell and Bakker, 2014; Herrera et al., 2015), differences between WL and SL can be less marked or even disappear.

At any rate, the degree of intraindividual variability depends on the number of leaf flushes during one growing season, which in turn is strongly affected by climate (Niinemets, 2014). These considerations become extremely important in a context of climatic changes which are not only expected to modify the length of the favorable period for growth of Mediterranean species (Correia and Ascensão, 2017), but also to generate stochastic changes of the thermal amplitudes over the year, with predictable consequences on leaf structuring and functioning.

Most of research involving seasonal dimorphic species has been focused on the differences between WL and SL. However, a shift in focus on the continuous effect of seasonal environmental changes on intraindividual variability of leaf traits in seasonal dimorphic species is strongly needed to provide new mechanistic insights on how these species thrive in the ever-changing climatic conditions typical of the Mediterranean climate.

## Author Contributions

The author confirms being the sole contributor of this work and has approved it for publication.

### Conflict of Interest Statement

The author declares that the research was conducted in the absence of any commercial or financial relationships that could be construed as a potential conflict of interest.
